# Glaukos iStent inject® Trabecular Micro-Bypass Implantation Associated with Cataract Surgery in Patients with Coexisting Cataract and Open-Angle Glaucoma or Ocular Hypertension: A Long-Term Study

**DOI:** 10.1155/2016/1056573

**Published:** 2016-11-01

**Authors:** Pedro Arriola-Villalobos, Jose Maria Martinez-de-la-Casa, David Diaz-Valle, Laura Morales-Fernandez, Cristina Fernandez-Perez, Julian Garcia-Feijoo

**Affiliations:** ^1^Servicio de Oftalmología, Hospital Clínico San Carlos, Instituto de Investigación Sanitaria del Hospital Clínico San Carlos (IdISSC), Madrid, Spain; ^2^Cooperative Research Network on Age-Related Ocular Pathology, Visual and Life Quality, Instituto de Salud Carlos III, Madrid, Spain; ^3^Servicio de Oftalmología, Hospital Clínico San Carlos, Departamento de Oftalmología y ORL, Facultad de Medicina, Universidad Complutense de Madrid, Instituto de Investigación Sanitaria del Hospital Clínico San Carlos (IdISSC), Madrid, Spain; ^4^Servicio de Medicina Preventiva y Salud Pública, Hospital Clínico San Carlos, Instituto de Investigación Sanitaria del Hospital Clínico San Carlos (IdISSC), Madrid, Spain; ^5^Instituto de Investigaciones Oftalmológicas Ramon Castroviejo, Universidad Complutense de Madrid, Madrid, Spain

## Abstract

*Purpose*. To evaluate the long-term efficacy and safety of the iStent* inject* device (Glaukos Corporation, Laguna Hills, CA) combined with phacoemulsification in patients with coexistent cataract and open-angle glaucoma or ocular hypertension (OHT).* Methods*. A prospective, uncontrolled, nonrandomized, interventional case series study was conducted in patients with both mild or moderate open-angle glaucoma or OHT and cataract. Patients underwent cataract surgery along with the implant of two iStent* inject* devices. Outcome measures were intraocular pressure (IOP), topical hypotensive medications required, and best-corrected visual acuity (BCVA).* Results*. 20 patients were enrolled. Mean follow-up was 47.4 ± 18.46 months. Mean baseline IOP was 19.95 ± 3.71 mmHg with medication and 26 ± 3.11 mmHg after washout. Mean end-follow-up IOP was 16.25 ± 1.99 mmHg, representing an IOP decrease of 36.92%, 9.74 ± 3.14 mmHg (*P* < 0.001), from baseline washout IOP. The mean number of medications was significantly reduced from 1.3 ± 0.66 to 0.75 ± 0.79 (*P* = 0.017). 45% of patients were medication-free by the end of follow-up. Mean log⁡MAR BCVA improved significantly from 0.42 ± 0.16 to 0.18 ± 0.16 (*P* < 0.001). No complications of surgery were observed.* Conclusion*. The iStent* inject* device combined with cataract surgery served to significantly reduce both IOP and medication use in the long term in patients with coexistent open-angle glaucoma or ocular hypertension (OHT) and cataract.

## 1. Introduction

Glaucoma is the leading cause of irreversible blindness worldwide and is estimated to currently affect more than 60 million people [1]. Elevated intraocular pressure (IOP) is the main risk factor for the onset and advance of glaucoma. In effect, reducing IOP is the accepted consensus strategy to delay or even avoid the development of glaucoma and to manage its progression [2]. Thus, the ideal treatment for glaucoma should offer continuous IOP management along with a favourable safety profile.

Filtration surgery is usually performed in moderate or advanced cases but is linked to complications [3] including visual loss, bleb leak, inflammation, hypotony, and endophthalmitis [4]. Microinvasive glaucoma surgery tries to preserve conjunctival tissue of scarring through an ab interno microincisional approach, with efficacy, high safety profile, and fast recovery in mild-to-moderate glaucoma [5]. The iStent Trabecular Micro-Bypass (Glaukos Corporation, Laguna Hills, CA, USA) bypasses the trabecular meshwork (TM), which is the major source of resistance to outflow in open-angle glaucoma. iStent has been successfully employed to increase outflow in human anterior segments in vitro [6] and in glaucoma patients [7].

The first-generation iStent, Model GTS-100, is a titanium L-shaped stent that has proved safe and effective in lowering IOP in patients with mild-to-moderate glaucoma [7–10], even in the long term [11]. This device has also been described to achieve an IOP reduction to less than 15 mmHg if two or more iStent devices are implanted during cataract surgery [12].

A second-generation new microscale stent recently developed, the iStent* inject* Trabecular Micro-Bypass Model GTS-400, also increased outflow facility in cultured human anterior eye segments [13]. So far, three case series have revealed the safety and efficacy of the iStent* inject* device [14–16]. Another randomized study compared outcomes of two iStent* inject* devices versus two ocular hypotensive agents [17], showing that the use of iStent* inject* device is at least as effective as two medications. However, the follow-up of these studies was no longer than 12 months.

The purpose of our study was to assess the long-term efficacy and safety of implanting of two iStent* inject* Model GTS-400 stents combined with phacoemulsification in patients with coexistent cataract and open-angle glaucoma or ocular hypertension (OHT). This study is a continuation of our initial work [14], in which we reported outcomes one year after iStent inject device placement inserted via first-generation prototype G2-0 injectors.

## 2. Methods

Twenty patients with cataract and primary open-angle and pseudoexfoliative glaucoma or OHT were enrolled for this prospective, noncomparative, uncontrolled, nonrandomized, intervention study.

The study protocol adhered to the tenets of the Declaration of Helsinki and Spanish legislation and was approved by our Institutional Review Board. Before recruitment, written legally binding informed consent for Glaukos iStent* inject* implantation and cataract surgery was obtained from each patient.

Detailed subject inclusion criteria and surgical technique were previously described [14]. The main inclusion criteria were a previous diagnosis of mild-to-moderate open-angle glaucoma (including pseudoexfoliative glaucoma) or OHT and an IOP of 14–30 mmHg as measured at the last two consecutive visits if receiving ocular hypotensive medications, of 22–30 mmHg if not, or of 22–32 mmHg after hypotensive drops washout.

Before surgery, all the patients receiving ocular hypotensive medications were instructed to discontinue their use to obtain baseline IOP measurements. All surgical procedures were performed by two of the authors (JMMC and JGF), both with similar experience in MIGS surgery. Surgical technique is similar to that described for the GTS-100 device [13, 14]. After phacoemulsification and IOL placement, two GTS-400 iStent devices were implanted through the clear corneal incision (approximately 2.85 mm) placed for phacoemulsification. First-generation prototype G2-0 injectors were used to deliver the stents. Antiglaucoma topical therapy was introduced postoperatively if the desired target IOP range, as judged by the investigator, was not achieved.

Postoperative visits were scheduled for 1 day, 1 week, 1, 3, and 6 months, and 1, 2, 3, 4, 5, and 6 years after surgery. Every visit included slit-lamp biomicroscopy, applanation tonometer (Clement Clarke Perkins MK2 Tonometer) IOP measurement, number of glaucoma medications, and BCVA. Preoperatively and one month, three months, and every year postoperatively, the nasal angle was examined by gonioscopy.

### 2.1. Data Analysis

Efficacy outcome measures were IOP and topical ocular hypotensive medications used pre- and postoperatively. Successful treatment was defined as an IOP reduction ≥ 20% regardless of medication. We also defined “complete success” as an IOP ≤ 18 mmHg without postoperative medication and “qualified success” as an IOP ≤ 18 mmHg with medication [18]. These outcomes were determined by proportional analysis. Kaplan-Meier graphs were constructed to estimate surgical success according to those success criteria.

Failure rate included those patients who did not meet success criteria, required further glaucoma surgery, or had severe visual loss secondary to surgery itself.

Safety outcome measures were complications and log⁡MAR BCVA.

For a descriptive statistical analysis, we used Excel 2007 (Microsoft Corp.) with SPSS software (version 15.0, SPSS Inc.). Results are provided as the mean ± standard deviation (SD).

The Kolmogorov-Smirnov test was used to check the normal distribution of data. A paired-sample* t*-test was used to compare outcomes in the study group. Significance was set at *P* ≤ 0.05.

## 3. Results

Combined surgery was uneventful in all the participating patients and the iStent was successfully implanted in all eyes, although three patients received just one iStent for logistic reasons. Postoperatively, gonioscopy revealed the presence of only one iStent in a further four patients (20%). So, in seven patients (35%), only one iStent was confirmed as functional. Both groups were comparable in baseline IOP and meds data, because no significant differences were obtained at that time point.

### 3.1. Patient Demographics

Twenty Caucasian subjects (11 women) were enrolled in the study, eight of whom had primary open-angle glaucoma, eight had OHT, and four had pseudoexfoliative glaucoma.

Mean follow-up was 47.4 ± 18.46 months (range 12–72 months) and median follow-up was 60 months. Eleven of the patients completed at least five years of follow-up, and two of them were followed six years (Table 1). Nine patients did not complete five years visit, due to logistic reasons (*n* = 4) and mortality (*n* = 5).

### 3.2. IOP Results

**Figure 1:**
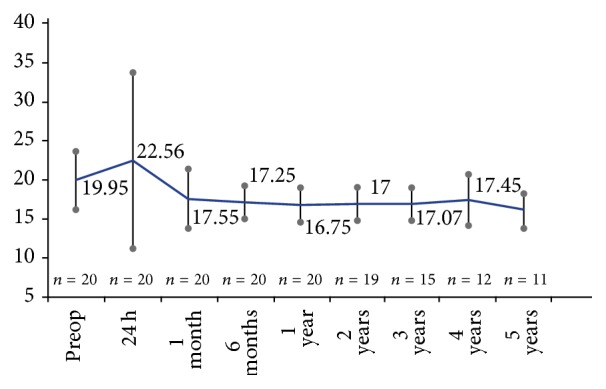
Mean intraocular pressure (IOP) (±standard error of the mean) recorded at each follow-up visit (baseline IOP is medicated IOP).

**Figure 2:**
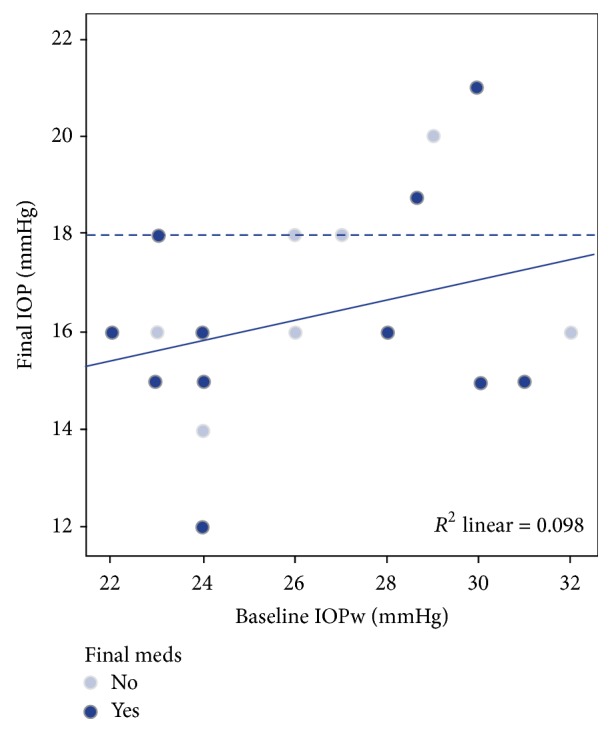
Scatter plot representing preoperative intraocular pressure (IOP) after washout and postoperative IOP without and with meds. Continuous line represents regression line (*R* ^2^ = 0.098). Discontinuous line represents IOP of 18 mmHg, defined as success in the study.

Mean IOP with medication at baseline was 19.95 ± 3.71 mmHg and after washout was 26 ± 3.11 mmHg (Figure 1). At the end of follow-up, mean IOP was 16.25 ± 1.99 mmHg. The IOP decrease from preoperative washout IOP was 9.74 ± 3.14 mmHg, representing a significant decrease of 36.92% (*P* < 0.001). The mean IOP drop relative to preoperative IOP with medication was 3.7 ± 3.7 mmHg, representing a 16.49% decrease (*P* < 0.001).  Figure 2 shows preoperative IOP and final IOP without and with medication in each patient via scatter plot (Figure 2).

In the 11 patients completing at least five years of follow-up, mean IOP with medication at baseline was 20.36 ± 4.57 mmHg and after washout was 25.7 ± 3.06 mmHg. End-follow-up mean IOP was 16.18 ± 2.27 mmHg. Reductions in IOP from preoperative washout IOP and from preoperative IOP with meds (9.5 ± 3.1 mmHg and 4.18 ± 4.62 mmHg; 36.56% and 17.52%; *P* < 0.001 and *P* = 0.013, resp.) recorded in this subset of 11 eyes were similar to the reduction observed for the entire set of 20 eyes.

Subanalysis of IOP results depending of the number of functional iStent devices showed no significant differences in final IOP decrease between one and two functional iStent devices (*P* = 0.425). However, the group with two iStent devices had an IOP decrease of 10.42 ± 3.6 mmHg versus 8.57 ± 1.81 mmHg in the one functioning iStent group.

Three eyes (15%) showed transient IOP elevation to above 30 mmHg one day postoperatively. We previously attributed this to retained viscoelastic and observed its resolution by the one-week visit [14]. There were no additional reports of IOP elevation during the remaining course of long-term follow-up.

### 3.3. Antiglaucoma Medications

**Figure 3:**
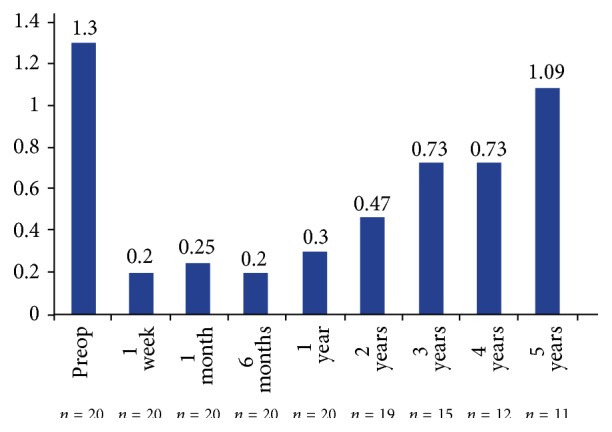
Use of glaucoma medications at each follow-up visit.

The majority of subjects reduced their medication burden during the course of follow-up. Before surgery, the mean number of glaucoma medications was 1.3 ± 0.66 (Figure 3). At the end of follow-up, the mean number of medications decreased to 0.75 ± 0.79, representing a significant mean reduction in glaucoma medications of 0.5 ± 0.89 (*P* = 0.017).  Table 2 shows the proportion and number of patients receiving no, one, or two medications at baseline and at the final of follow-up.

At one year, 15 patients (75%) required no topical therapy, while just one subject (5%) needed two antiglaucoma medications. At the 3-year visit, seven of the 13 patients (53.9%) who completed the three years of follow-up were using one or two medications, while six patients were medication-free (46.2%). At five years, three patients (27.3%) were medication-free; four patients (36.4%) were taking one medication, while four patients (36.4%) were using two antiglaucoma drops. In the 11 eyes with 5-year follow-up data, the number of subjects on two medications translated to a nonsignificant glaucoma medication reduction in that group of patients.

The mean number of medications administered preoperatively in the patients who completed five years of follow-up was 1.18 ± 0.75. At the end of follow-up, this figure fell to 1.09 ± 0.83 medications, representing a mean reduction of 0.09 ± 0.94 (*P* = 0.756). At five years, three of the 11 patients (27.3%) were free of medication.

Subanalysis of medication use results depending of the number of functional iStent devices showed no significant differences in the final mean number of glaucoma medications (*P* = 0.255). However, the group with two iStent devices had a mean reduction of 0.77 ± 0.93 meds versus 0.14 ± 0.9 meds in the one functional iStent group. Besides, the percentage of eyes not requiring antiglaucomatous medications at the end of follow-up was higher in the group with two functioning iStent devices (61.5%) than in the group with one functioning iStent (14.3%).

### 3.4. Success Rate

At three years of follow-up, 33% of patients achieved an unmedicated IOP reduction ≥ 20% versus baseline unmedicated IOP, and 86% experienced an IOP reduction ≥ 20% regardless of medication. At five years, the percentage of patients showing an unmedicated IOP reduction ≥ 20% versus baseline washout IOP was 20%, while 100% experienced an IOP reduction ≥ 20% regardless of medication. At this follow-up time, seven out of 11 patients (63.63%) showed an IOP ≤ 16 mmHg regardless of medication.

**Figure 4:**
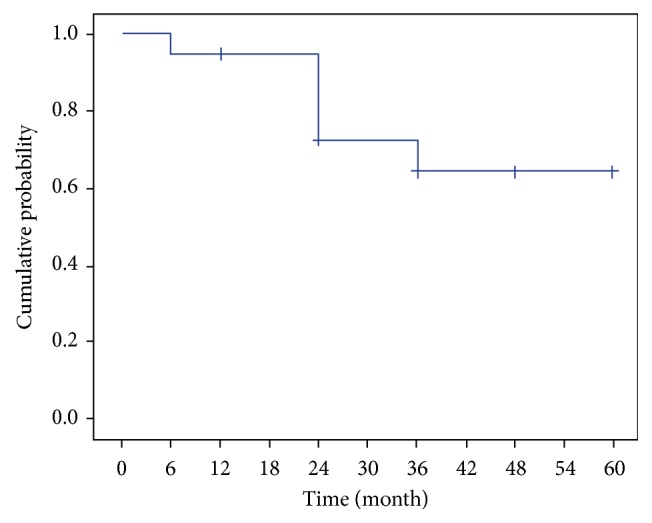
Kaplan-Meier plot of the cumulative probability of success defined as an IOP reduction ≥ 20% regardless of medication.

The cumulative probability of success defined as an IOP reduction ≥ 20% using Kaplan-Meier survival analysis was 72.4% at two years and 64.4% at three years (Figure 4).

**Figure 5:**
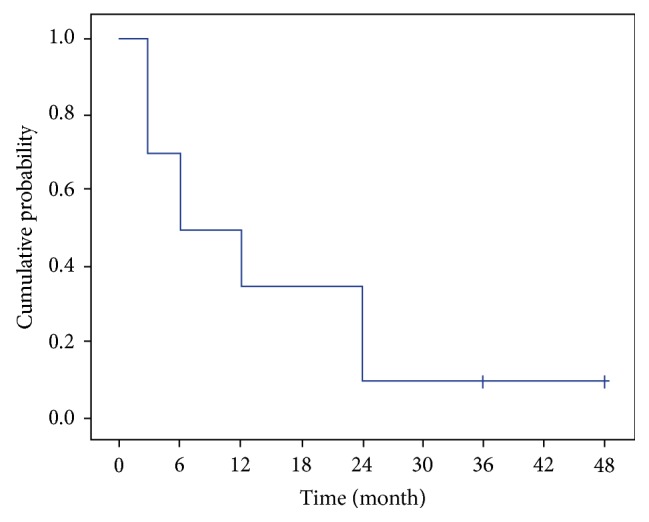
Kaplan-Meier plot of the cumulative probability of complete success, defined as an IOP ≤ 18 mmHg without postoperative medication.

**Figure 6:**
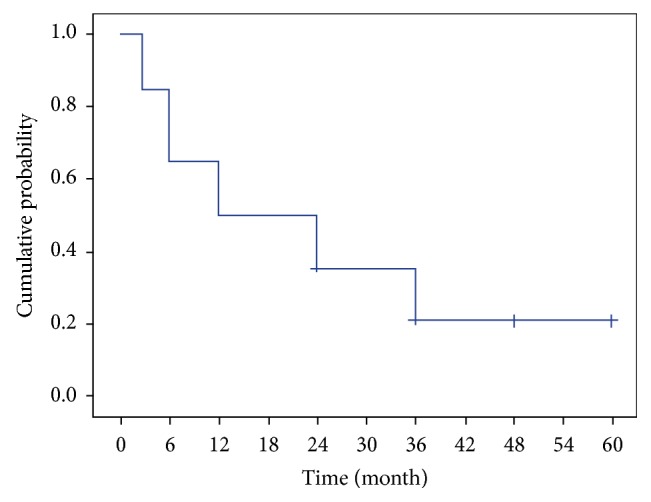
Kaplan-Meier plot of the cumulative probability of “qualified success,” defined as an IOP ≤ 18 mmHg with medication.

Complete success at the end of follow-up was recorded in eight patients (40%) and qualified success in 10 of our 20 patients (50%). Similar rates were obtained for the subset of patients completing five years of follow-up, though the complete success rate was slightly lower and the qualified success higher (Table 3). The cumulative probability of complete and qualified success using Kaplan-Meier survival analysis at two years was 10% and 35%, respectively (Figures 5 and 6).

By the end of follow-up, 15 of the 20 subjects (75%) showed an IOP ≤ 16 mmHg regardless of medication, including six patients of the 20 (30%) requiring no medication.

No patient needed any further glaucoma surgery over the follow-up period.

### 3.5. Safety Outcomes

No visual acuity loss was recorded; log⁡MAR BCVA significantly improved from a mean of 0.42 ± 0.16 preoperatively to 0.18 ± 0.16 at the end of follow-up (*P* < 0.001).

No adverse events related to iStent* inject* implantation were reported. During the long-term follow-up, five patients suffered an eye condition unrelated to iStent* inject* implantation. Three of these patients required Nd:YAG capsulotomy due to posterior capsule opacification (PCO). One patient (who had OHT and high myopia) suffered retinal detachment 40 months after the initial surgery, with excellent visual recovery after pars plana vitrectomy. The last of these patients developed epiretinal membrane three years after the initial procedure. However, no surgery was required due to the patients' lack of symptoms and good visual acuity.

## 4. Discussion 

In this series of 20 patients, the implant of two iStent* inject *devices using first-generation prototype G2-0 injectors combined with phacoemulsification led to a significant IOP reduction (*P* < 0.001) and a significant decrease in the number of antiglaucoma medications required (*P* = 0.017) after a mean follow-up of almost four years. The subset of 11 patients completing five years of follow-up showed a similar significant IOP decrease (*P* < 0.001) along with a nonsignificant decrease in the number of antiglaucoma medications required (*P* = 0.756).

In both the full set of 20 eyes and the subset of 11 eyes, the IOP reduction from medicated preoperative IOP was approximately 4 mmHg. This decrease is higher than the approximate 2 mmHg decrease reported after cataract surgery alone [19].

IOP reduction in this small patient cohort was not as pronounced as that reported in the larger studies that have addressed iStent* inject* implantation (Table 4) as a single procedure [15–17]. In all these studies, two iStent* inject* devices were implanted per eye. This difference in outcome may be attributed to shorter follow-up times. Our study outcomes may have been affected by the fact that the patients in this trial were the first at our centre to receive an iStent* inject* device such that surgeons were at the start of their learning curve. Further, we used first-generation prototype G2-0 injectors in all 20 patients in this series although a second-generation injector (G2-M-IS) has since been introduced. This new model is able to hold two stents so that the clinician can insert two devices while entering the eye only once. This improved injector design reduces the number of surgical steps and thus raises the chances of more reliable surgery and improved outcomes. Further, in our prior study, in a large proportion of patients (7/20), only one functional stent was observed on postsurgery gonioscopy [14]. It is likely that these patients would have shown a greater IOP reduction if two stents had been confirmed as functioning, based on in vitro results indicating that that a second stent achieved a further increase in outflow facility [4]. In effect, Belovay et al. [12] observed the improved ability of multiple GTS-100 iStent devices to reduce IOP to below 15 mmHg and reduce topical ocular hypotensive medications. In fact, our subgroup analysis, although not significant, showed higher decrease in IOP and medication use at the end of the follow-up in the group of two functioning iStent devices. These differences could be significant with higher sample size.

In our patients, IOP was stable during follow-up (Figure 1). The number of medications used increased over time after the one-year visit (Figure 3). A slight increase in the number of medications beyond one year of follow-up was also reported in the long-term study in which one iStent GTS-100 was implanted during phacoemulsification [11]. Notwithstanding, a discrete reduction was produced in the number of antiglaucoma drops at five years, including three patients (27.3%) who were medication-free. A recent report has shown that the placement of two iStent GTS-400 devices as a sole procedure is at least as effective as two medications [17]. Reducing or eliminating the use of antiglaucoma drops is highly desirable because the chronic use of antiglaucoma drugs may lead to ocular surface damage and conjunctival inflammation [20] or even reduce the success of subsequent trabeculectomy [21].

Success in our study defined as an IOP reduction ≥ 20% regardless of medication was achieved in all the patients at the end of follow-up. At that time point, an IOP reduction ≥ 20% with no medications was achieved in nine out of 20 patients, to give a success rate of 45%. Beyond the one-year visit, success rates fell (Table 2), consistent with the increased use of antiglaucoma medications. We hypothesize that ultrastructural changes in the TM or Schlemm's canal could be responsible for this increase in medications required. Our complete success rate at one year (50%) was lower than the other reported rates for two GTS-400 devices at one year. Fea et al. [17] reported a 92.6% of complete success, while Voskanyan et al. [15] reported 66%. Probable reasons for this discrepancy have been analysed above.

Our findings indicated a highly acceptable safety profile, with no adverse events or long-term complications related to stent implant. No subjects experienced hypotony, endophthalmitis, or sight-threatening complications associated with more invasive surgery procedures. Mean visual acuity was significantly improved at each follow-up visit. There was a mild decrease in mean visual acuity beyond the one-year visit, attributed mainly to PCO.

Our study has several limitations. The number of patients was low and, being a long-term study, several patients were lost to follow-up such that only slightly more than half of the patients completed 60 months of follow-up. Being uncontrolled, we could not determine the individual effects of phacoemulsification or iStent placement on IOP and the number of hypotensive drugs required. The cohort examined was a heterogeneous group of subjects, with mild-moderate primary open-angle or pseudoexfoliative glaucoma along with OHT patients. Moreover, as reported above, these patients were the first to receive the iStent GTS-400 at our centre, meaning that, despite experience with the GTS-100 model, the surgeons were at an early learning stage with the use of the new model, which could affect outcomes. Finally, in seven patients (35%), only one iStent was confirmed as functional [14]. This potential shortcoming could also affect the IOP and medication use results [7, 12], although nonsignificant differences were found.

## 5. Conclusions

To the best of our knowledge, this is the first study to assess combined GTS-400 iStent implantation and phacoemulsification surgery over a follow-up period longer than one year. Significant reductions were achieved in IOP and number of medications required after more than 47 months of follow-up. At the end of follow-up, 45% of the patients were medication-free and all patients showed good visual outcomes with no serious adverse events recorded. Our findings suggest that iStent GTS-400 placement added to phacoemulsification could be a long-term safe and effective treatment option alternative for patients with both cataract and mild-moderate open-angle glaucoma or OHT. These findings, nevertheless, require confirmation in randomized controlled studies conducted in large patient cohorts.

## Figures and Tables

**Table 1:** Postoperative follow-up of 20 patients (*n*: number of patients).

	Surgery	1 year	2 years	3 years	4 years	5 years	6 years
*n*	20	20	19	15	12	11	2

**Table 2:** Number of antiglaucoma medications used at baseline and final follow-up.

Follow-up time	None drops	One drop	Two drops
Baseline	2 (10%)	10 (50%)	8 (40%)
Final	9 (45%)	7 (35%)	4 (20%)

**Table 3:** Success rates recorded during follow-up (w/o: without).

Follow-up time	IOP reduction ≥ 20%		Complete success	Qualified success
	w/o meds	with or w/o meds		
1 month	63%	84%	55%	10%
3 months	84%	100%	65%	15%
6 months	73%	94%	50%	15%
1 year	73%	100%	50%	20%
2 years	25%	62%	35%	41%
3 years	33%	86%	31%	46%
4 years	30%	80%	28%	36%
5 years	20%	100%	28%	64%

**Table 4:** Outcome variables reported in the studies that have addressed the efficacy of iStent GTS-400 placement. The data provided for the present study are end-follow-up data for the 20 eyes enrolled and 5-year results for 11 eyes.

Source	*n*	Combined phaco	Follow-up (months)	Mean IOP reduction (mmHg) from preop	IOP ≤ 18 mmHg w/o meds	Mean reduction in meds
Arriola-Villalobos et al. [14]^*∗*^	20	Yes	12	3.2 ± 3.75	50%	1 ± 0.79
Voskanyan et al. [15]	92	No	12	22.1 ± 3.3 versus 15.7 ± 3.7^†^	66%	NA
Klamann et al. [16]	17	No	6	21.19 ± 2.56 versus 14.19 ± 1.38^†^	NA	2.19 ± 0.91 versus 0.88 ± 0.62^†^
Fea et al. [17]	94	No	12	8.1 ± 2.6	92.6%	NA
Present study^*∗*^	20	Yes	47.4 ± 18.46	3.7 ± 3.7	40%	0.6 ± 0.88
	11		60	4.18 ± 4.62	28%	0.09 ± 0.94

Preoperative IOP refers to medicated IOP. (*n*: number of patients; w/o: without; ^†^data not available, mean preoperative versus mean postoperative data; ^*∗*^same set of patients; NA: not available.)
